# Supplementary material for: Small-Group Discussion Sessions on Imposter Syndrome
Source: MedEdPORTAL. 2020 Nov 10;16:11004. doi: 10.15766/mep_2374-8265.11004 (PMC7666839; doi:10.15766/mep_2374-8265.11004)
Supplement: Supplementary file 1 — Imposter Syndrome Facilitator Guide.docxImposter Syndrome Handout.docxImposter Syndrome Survey.docx [file mep_2374-8265.11004-s001.zip › A. Imposter Syndrome Facilitator Guide.docx]

**Imposter Syndrome Facilitator Guide**

**Objectives**

After completing this session, participants will be able to:

1. Define imposter syndrome and ways it can manifest
2. Identify risk factors associated with imposter syndrome
3. Develop strategies to overcome imposter syndrome

**Information for facilitators to read prior to leading group discussion**

**Introduction**

- Imposter syndrome is prevalent in medical students and residents^1,2^ and has been linked to an increased incidence of burnout^2,3^
  - Occurs in people who often have been very successful but are afraid that their success is due to luck or hard work
  - Characterized by chronic feelings of self-doubt and fear that other people will “find out” that they are not worthy of their accomplishments

**Causes of imposter syndrome**

- Imposter syndrome often occurs in high achieving individuals because this outward recognition of accomplishments leads to the feeling that one cannot live up to expectations
- Physicians are at high risk for imposter syndrome because they are surrounded by other high achieving individuals and lose sight of their own achievements by comparing themselves with peers^4^
- Imposter syndrome is twice as common in women and more common in international medical graduates^2,3,5^
- Imposter syndrome is thought to be more common at times of transition into new roles. However, it can occur at all levels of training and does not necessarily abate over time or with continued success^4,6^

**Strategies to alleviate imposter syndrome**

- Strategies for alleviating imposter syndrome include the following^4^:
  - Recognize that the feelings of inadequacy are not unusual
  - Avoid maladaptive strategies such as perfectionism and procrastination
  - Pay attention to external objective evidence of success and positive feedback from peers
  - Practice self-compassion when an error or perceived failure occurs
- Increasing diversity and promoting gender equality in the workplace are also essential^5^

**Instructions for facilitating discussion**

This is designed as a highly interactive session for trainee physicians. Ideally the number of participants in this discussion would be between 5 and 10. If you anticipate more participants, breaking into small group discussions or pairing up to discuss a prompt can allow for comfort in a larger group setting**.**The session can be led by one facilitator. If possible, it is helpful to have a chief resident or attending physician in attendance to jump start discussion and normalize the feelings of imposter syndrome. Be vulnerable - share your experiences with self-doubt and failure so that participants feel safe sharing their stories, especially if there is reluctance to participate. Consider starting the session with an ice-breaker to allow participants to introduce themselves to one another. Make sure to participate in the ice-breaker so that participants feel more comfortable with you.

The agenda described below contains content about the definition and rates of imposter syndrome along with numerous discussion-prompting questions. Some questions are to be posed to the entire group. Other activities are modeled as a “think-pair-share.” In a “think-pair-share” activity, participants are asked to pair up and discuss the prompt question with their partner. The learning environment should allow participants to share personal experiences during think-pair-share privately without fear of others overhearing. Subsequently, the facilitator will ask for volunteers to share with the group. Some sections of the curriculum may be challenging for some participants to discuss. If there appears to be a lack of participation, allowing for silence of at least 10 seconds is encouraged to provide opportunity for participants to reflect, collect and share their thoughts on this sensitive subject. Do not rush to break the silence. It is also helpful to ask the chief resident or attending physician at the session to speak up, answer questions, or describe an experience if participants are otherwise not participating. Be an active listener by using non-verbal cues such as head nodding and eye contact, reflecting back what you understand about what was shared and boomeranging questions back to the group. Acknowledge the feelings and experiences shared in the group through mirroring with statements such as "thank you for sharing that with the group" and "that must have been a difficult situation to be in." Ask participants to clarify questions or statements that are difficult to understand. Try not to make assumptions. Refocus the discussion when needed.

Individuals may be at higher risk for imposter syndrome based on their identified or perceived race or gender due to systemic racism and sexism. As a result, issues related to gender, ethnicity, or race may be brought up by participants. The facilitator should begin the session by setting ground rules for open and respectful discussion. These discussions may raise uncomfortable feelings or conflicts, but the facilitator should emphasize that our goal is to create a safe space where we respect one another and all opinions are heard. All discussion participants should realize that when you share your opinion, you are speaking only for yourself. Never presume to ask someone to speak on behalf others who share the same race, gender, or sexual orientation. Finally, the group should be reminded that our discussion space is confidential. While concepts and ideas can leave the group, personal details must not.

In our experience, there are no “frequently-asked-questions” as participants report understanding the content presented since the focus of the session is having an open discussion. In most wellness sessions, participants express concerns about lack of time or resources to engage in more self-care. It would be beneficial to acknowledge the concern and state that while systemic change is needed to fully address wellness, we are also offering education and an opportunity to learns skills to address the needs on a more individual level.

**Session Agenda**

| 5 minutes (10 minutes if including optional icebreaker)  Facilitator | Facilitator welcomes participants and reviews objectives. Consider starting the session with an ice-breaker to allow participants to introduce themselves to one another.  Prior to beginning session, facilitator will acknowledge difficulty of the subject, set ground rules for discussion and ask participants to recognize their implicit biases and create a supportive space for discussion.   - These discussions may raise uncomfortable feelings or conflicts, but our goal is to create a safe space where we respect one another and all opinions are heard. - Realize that when you share your opinion you are speaking only for yourself. Never presume to ask someone to speak on behalf others who share the same race, gender, or sexual orientation. - While concepts and ideas can leave the group, personal details must not.   Facilitator will then define imposter syndrome:   - Imposter syndrome is characterized by chronic feelings of self-doubt and fear of being discovered as a fraud. - Often people who have been very successful by external standards feel their success has been due to luck or great effort. They do not think that their success is the result of their own ability and competence. - They are often afraid they will be unable to duplicate their success when a new task arises.   Facilitator will review prevalence in medical trainees   - Medical students: 49% of women, 23% of men^2^ - Family medicine residents: 33% (41% women, 24% men)^1^ - More prevalent in international medical graduates (OR 10.7)^3^ |
| --- | --- |
| 10 minutes  Facilitator and Participants  +/- Chief resident or attending | Facilitator will pose discussion questions to participants:  Why are physicians at risk for imposter syndrome?   - High achieving individuals surrounded by other high achieving individuals with whom they compare themselves - Women physicians, international medical graduates and underrepresented minority physicians are at particular risk due to the presence of systemic racism and sexism   What environmental factors lead to imposter syndrome?   - Transitions, new experiences (clinically or professionally) at any level of training - Implicit and explicit biases of colleagues - Presence of structural racism and sexism   What personality traits lead to imposter syndrome?   - Perfectionism - “Type A” personality - High achieving   Do you think imposter syndrome is a state or a trait?   - Most participants will identify that imposter syndrome is a state which exists transiently and can be overcome by various strategies. However, there may be some participants who have chronic feelings of imposter syndrome such that they feel it is an inherent trait.   Why are women at risk for imposter syndrome?   - Penalized for exercising power, being opinionated or overly ambitious 🡪 conditioned to be humble, accommodating, self-deprecating - Lack of female role models in leadership positions |
| Think-Pair-Share: 7 minutes  Facilitator and Participant Pairs | Participants will break into pairs and participate in a “think-pair-share” exercise. This is a cooperative learning strategy that encourages self-reflection and provides the opportunity to communicate one’s feelings with a peer and ultimately, the larger group. Facilitator will ask participants to think individually about a time during residency where they felt imposter syndrome. Individuals will then discuss this with their partners. Finally, pairs are asked to share their experiences with the larger group to facilitate further discussion.  Potential responses:   - Codes or ACTs - Breaking bad news - Missing key history or physical exam findings - First day of floors/ICU - First day as senior resident - Giving a presentation in front of peers |
| 3 minutes  Facilitator | Facilitator will discuss the presence of imposter syndrome outside of the medical field using the following quotes:   - Tom Hanks: “No matter what we’ve done, there comes a point where you think, ‘How did I get here? When are they going to discover that I am, in fact, a fraud and take everything away from me?’”^7^ - Dr. Margaret Chan, former Director-General of the World Health Organization: “There are an awful lot of people out there who think I’m an expert. How do these people believe all this about me? I’m so much aware of all the things I don’t know.”^8^ - Justice Sonia Sotomayor: “I’m not a classic imposter syndrome person because I have that initial insecurity but I’m capable of stepping outside of it and proving to myself it’s wrong.”^9^ |
| 5 minutes  Facilitator and Participants | Facilitator will discuss strategies for overcoming imposter syndrome^4^ and encourage participants to provide feedback on strategies they have successfully used in the past.   - Recognize negative thoughts and shift your focus to your successes. - Acknowledge your expertise and compare yourself with junior colleagues to see how much you have grown. - Remember what you do well. - Talk to your mentors and get feedback. - Reach out to your peers and find out how common this is - Practice realistic appraisal. Realize no one is perfect. - Practice self-compassion – think about how you would respond to a friend who approached you with the same situation. - Watch out for cycles of procrastinating or overpreparing. |
| Individual Reflection Exercise: 7 minutes  Facilitator and Participants  +/- Chief resident or attending | Facilitator will provide handouts (Appendix B) and offer the following prompts for participants to reflect on individually:   1. Write down a time when you worried about failing and instead succeeded. At the end of the story write the positive trait about yourself that helped you succeed in the task.   Responses regarding types of successes will vary.  Potential responses for positive traits:   - Resilience or flexibility - Being calm under pressure - Hardworking - Confident  1. How do you pump yourself up for a task you are worried about?   Potential responses:   - Reflect on your prior successes - Envision yourself successfully performing the task at hand - Talk with a close friend, family member or mentor - Listen to empowering music   Encourage participants to write down responses on handout. Ask for volunteers to share answers. |
| 3 minutes  Facilitator | Facilitator will discuss what to do if a participant has feelings of imposter syndrome or if a participant notices symptoms of imposter syndrome in a colleague   - Share your experiences with a peer – you might be surprised to find out they feel the same way - Reach out to a senior resident or faculty member - Seek support from the Employee Assistance Program (provide brochures or links to access the institution’s EAP) |
| 5 minutes  Facilitator and Participants | Conclusion: Ask participants to share one take away point from the session.  Survey distribution |

**References**

1 Oriel K, Plane MB, Mundt M. “Family medicine residents and the imposter phenomenon.” Family Medicine. 2004 Apr; 36 (4): 248-52.

2 Villwock J, Sobin LB, Koester LA, et al. “Imposter syndrome and burnout among American medical students: a pilot study.” International Journal of Medical Education. 2016; 7: 364-369.

^3^ Legassie J, Zibrowski EM & Goldszmidt MA. “Measuring Resident Well-Being: Impostorism and Burnout Syndrome in Residency.” J Gen Intern Med (2008) 23: 1090.

^4^ Chandra S, Huebert C, Crowley E, Das A. “Impostor Syndrome: Could it be Holding You or Your Mentees Back?” Chest. 2019 156(1): 26-32.

^5^ Mullangi S, Jagsi R. “Imposter Syndrome: Treat the Cause, Not the Symptom.” JAMA. 2019; 322(5):403–404.

^6^ LaDonna K, Shiphra G, Watling C. “’Rising to the Level of Your Incompetence’: What physicians self-assessment of their performance reveals about the imposter syndrome in medicine.” Acad Med. 2018;93(5):763-768.

^7^Tom Hanks says self-doubt is 'a high-wire act that we all walk; 2016. Available at: https://www.npr.org/2016/04/26/475573489/tom-hanks-says-self-doubt-is-a-high-wire-act-that-we-all-walk.  Accessed June 19, 2019.

^8^Pinker S. The Sexual Paradox: Troubled Boys, Gifted Girls and the Real Difference Between the Sexes.  New York: Scribner; 2008.

^9^Bravin, J. Memoir Details Justice's Difficult Ascent. The Wall Street Journal. Jan 14, 2013. Available at:

https://www.wsj.com/articles/SB10001424127887324595704578239760608699742. Accessed June 19, 2019.
