# Supplementary material for: Small-Group Discussion Sessions on Imposter Syndrome
Source: MedEdPORTAL. 2020 Nov 10;16:11004. doi: 10.15766/mep_2374-8265.11004 (PMC7666839; doi:10.15766/mep_2374-8265.11004)
Supplement: Supplementary file 1 — Imposter Syndrome Facilitator Guide.docxImposter Syndrome Handout.docxImposter Syndrome Survey.docx [file mep_2374-8265.11004-s001.zip › B. Imposter Syndrome Handout.docx]

**Imposter Syndrome: Handout**

Objectives:

Define imposter syndrome and ways it can manifest

Identify risk factors associated with imposter syndrome

Develop strategies to overcome imposter syndrome

Relevant Quotes from Highly Successful People about Imposter Syndrome:

- Tom Hanks, actor: “No matter what we’ve done, there comes a point where you think, ‘How did I get here? When are they going to discover that I am, in fact, a fraud and take everything away from me?’”^1^
- Dr. Margaret Chan, former Director-General, World Health Organization: “There are an awful lot of people out there who think I’m an expert. How do these people believe all this about me? I’m so much aware of all the things I don’t know.”^2^
- Sonia Sotomayor, Supreme Court Justice: “I’m not a classic imposter syndrome person because I have that initial insecurity but I’m capable of stepping outside of it and proving to myself it’s wrong.”^3^

Strategies to Combat Imposter Syndrome^4^:

- Change your thinking
  - Remember what you do well
  - Recognize negative thoughts and shift your focus to your successes
  - Acknowledge your expertise - compare yourself with junior colleagues to see how much you have grown
- Talk to your mentors and get objective, truthful and constructive feedback
- Practice self-compassion
  - Realize no one is perfect and that you are not alone
  - Practice realistic appraisal – think of how you would respond to a friend
- Watch out for vicious cycles of procrastinating or overpreparing

**References**

1’Tom Hanks says self-doubt is 'a high-wire act that we all walk;' 2016. Available at: https://www.npr.org/2016/04/26/475573489/tom-hanks-says-self-doubt-is-a-high-wire-act-that-we-all-walk.  Accessed June 19, 2019.

2Pinker S. The Sexual Paradox: Troubled Boys, Gifted Girls and the Real Difference Between the Sexes.  New York: Scribner; 2008.

3Bravin, J. Memoir Details Justice's Difficult Ascent. The Wall Street Journal. Jan 14, 2013. Available at:

https://www.wsj.com/articles/SB10001424127887324595704578239760608699742. Accessed June 19, 2019.

^4^ Chandra S, Huebert C, Crowley E, Das A. “Impostor Syndrome: Could it be Holding You or Your Mentees Back?” Chest. 2019 156(1): 26-32.
