# Supplementary material for: Small-Group Discussion Sessions on Imposter Syndrome
Source: MedEdPORTAL. 2020 Nov 10;16:11004. doi: 10.15766/mep_2374-8265.11004 (PMC7666839; doi:10.15766/mep_2374-8265.11004)
Supplement: Supplementary file 1 — Imposter Syndrome Facilitator Guide.docxImposter Syndrome Handout.docxImposter Syndrome Survey.docx [file mep_2374-8265.11004-s001.zip › C. Imposter Syndrome Survey.docx]

| Imposter Syndrome | | | | | |
| --- | --- | --- | --- | --- | --- |
| Question | Strongly disagree | Disagree | Neutral | Agree | Strongly agree |
| This was an effective intervention to promote resident wellness. |  |  |  |  |  |
| The facilitator was helpful in fostering discussions. |  |  |  |  |  |
| I feel comfortable recognizing imposter syndrome in myself. |  |  |  |  |  |
| I feel comfortable recognizing imposter syndrome in my colleagues. |  |  |  |  |  |
| I feel comfortable discussing imposter syndrome with my colleagues. |  |  |  |  |  |
| I know what to do next if myself or my colleague has imposter syndrome. |  |  |  |  |  |

Free text:

**What did you like the most about wellness report?**

**What can we do to improve future wellness reports such as this?**

**Do you have suggestions for future wellness reports?**
