# Supplementary material for: Small-Group Discussion Sessions on Imposter Syndrome
Source: MedEdPORTAL. 2020 Nov 10;16:11004. doi: 10.15766/mep_2374-8265.11004 (PMC7666839; doi:10.15766/mep_2374-8265.11004)
Supplement: Supplementary file 1 — Imposter Syndrome Facilitator Guide.docxImposter Syndrome Handout.docxImposter Syndrome Survey.docx [file mep_2374-8265.11004-s001.zip › _Educational_Summary_Report_11004.pdf]

MedEdPORTAL® The AAMC Journal of Teaching and Learning Resources

Original Publication Open Access

Small-Group Discussion Sessions on Imposter Syndrome

Natalie Baumann, MD*, Carol Faulk, MD, Jessica Vanderlan, PhD, Justin Chen, MD, Rakhee K. Bhayani, MD

*Corresponding author: natalie.m.baumann@gmail.com

Abstract

Introduction: The Accreditation Council for Graduate Medical Education requires residency programs to support residents' well-being via established policies and programs. Imposter syndrome has been linked to burnout in residents, and understanding how to combat it may help improve resiliency in residents. **Methods:** We held a facilitator-guided, interactive discussion session for internal medicine residents on the topic of imposter syndrome as part of a larger series of discussion sessions on resident wellness. We repeated the session to capture a different group of residents. A psychologist or chief resident led each 30- to 45-minute session with the option to include an attending physician. Residents, faculty, and a clinical psychologist developed instructions for leading this session. At the end of each session, the facilitator provided attendees with a handout with take-home points and an optional postsurvey to assess learning objectives and ask whether they felt this was an effective intervention to promote resident wellness. **Results:** We collected data from 21 residents who attended the small-group discussion sessions. Ninety-six percent of residents felt comfortable recognizing imposter syndrome in themselves, and 62% knew the appropriate next steps after identifying imposter syndrome. Eighty-one percent of residents felt that the imposter syndrome wellness session was an effective intervention to promote resident wellness. **Discussion:** Imposter syndrome has been linked to resident burnout, and discussing imposter syndrome was viewed as an effective intervention to promote resident wellness and resiliency. When creating wellness interventions, other programs should consider addressing imposter syndrome.

Keywords
Wellness, Impostor Syndrome, Impostor Phenomenon, Burnout, Residency, Well-Being/Mental Health, Self-Regulated Learning

Educational Objectives

After completing this curriculum, residents will be able to:

1. Define imposter syndrome and ways it can manifest.
2. Identify risk factors associated with imposter syndrome.
3. Develop strategies to overcome imposter syndrome.

Introduction

The Accreditation Council for Graduate Medical Education requires residency programs to establish "policies and programs that encourage optimal resident… well-being,"[1] which has led to an expanded focus on wellness topics throughout residency training. Imposter syndrome has been linked to burnout in residents,[2,3] and understanding how to combat imposter syndrome may help improve resiliency in residents. Clance and Imes first described the concept of imposter syndrome (also known as impostor syndrome or impostor phenomenon) in 1978.[4] Clance and Imes noted that many high-achieving women did not experience an internal feeling of success and in fact felt that their success was attributable to chance or luck. Women with imposter syndrome feared that their perceived incompetence would be discovered by those around them, leading to exposure as a fraud or imposter. These feelings were present despite earning multiple advanced degrees and receiving positive feedback from colleagues and superiors. While Clance and Imes focused their research on high-achieving nonphysician women, it is well known that imposter syndrome occurs in both male and female physicians. A 2016 study by Villwock, Sobin, Koester, and Harris of over 130 American medical students identified symptoms of imposter syndrome in roughly 49% of female students and 24% of male students.[2] Oriel, Plane, and Mundt found a very similar prevalence amongst family medicine residents, with 41% of women and 24% of men expressing symptoms consistent with imposter syndrome.[5] Feelings of self-doubt can occur at any point in a physician's career[6] but can be heightened during times of transition such as residency training. Not only does imposter syndrome cause emotional stress in the individual, but it has been linked to physician burnout.

Citation:
Baumann N, Faulk C, Vanderlan J, Chen J, Bhayani RK. Small-group discussion sessions on imposter syndrome. *MedEdPORTAL*. 2020;16:11004. https://doi.org/10.15766/mep_2374-8265.11004

Copyright © 2020 Baumann et al. This is an open-access publication distributed under the terms of the Creative Commons Attribution license.

Villwock and colleagues found that imposter syndrome was significantly associated with increased components of burnout, including exhaustion, emotional exhaustion, cynicism, and depersonalization.[2] A 2008 study by Legassie, Zibrowksi, and Goldszmidt also noted a negative correlation between feelings of imposter syndrome and personal achievement scores as measured by the Maslach Burnout Inventory amongst 48 internal medicine residents; international medical graduates in their study were more likely to have feelings of imposter syndrome with an odds ratio of 10.7.[3]

Understanding how to identify and combat imposter syndrome may help improve resiliency in residents and prevent the development of burnout. Previously described strategies to combat imposter syndrome include objectively evaluating one's successes and the skills needed to achieve them, seeking constructive feedback from trusted mentors, and practicing self-compassion.[7] There is limited educational material published regarding imposter syndrome in residency, and there are no publications on this topic in *MedEdPORTAL*. A 2019 article by Ramsey and Spencer discussed a 3-hour educational pilot session on imposter syndrome integrated into intern orientation week.[8] In these sessions, interns were placed in small groups with attendings and chief residents and asked to role-play scenarios where they might develop feelings of imposter syndrome. Our unique discussion-based curriculum aims to educate residents of all training levels on imposter syndrome, identify risk factors for imposter syndrome, and teach skills to combat these feelings in order to improve overall resident wellness.

Methods

Curricular Context

We held a facilitator-guided, interactive, small-group discussion session on the topic of imposter syndrome as part of a larger series of small-group discussion sessions on resident wellness. We conducted the session twice to capture different groups of residents. Our audience included internal medicine residents in all 3 years of training at a large academic medical center. Our facilitator guide (Appendix A) outlined instructions for leading this session and was developed by a group of residents, faculty, and a clinical psychologist. A psychologist or a chief resident led each 30- to 45-minute session with the option to include an attending physician for additional support. No prerequisite knowledge was necessary for attendees.

Implementation

To begin each session, we defined imposter syndrome and reviewed data on the presence of imposter syndrome in medical trainees. For the next 10 minutes, we posed multiple questions for group discussion such as "Why are physicians at risk for imposter syndrome?" and "What environmental factors or personality traits could lead to imposter syndrome?" We then encouraged participants to spend about 7 minutes to reflect on times when they felt symptoms of imposter syndrome and discuss with a partner using the think-pair-share model. This cooperative learning strategy encouraged self-reflection and provided the opportunity to communicate one's feelings with a peer and, ultimately, the larger group. We then transitioned discussion to the presence of imposter syndrome in fields outside of medicine, including the law, business, and entertainment industries.[9-11] We reviewed strategies for managing imposter syndrome, such as seeking advice and feedback from mentors, discussing with peers, avoiding overpreparing and procrastination, practicing self-compassion, and recognizing one's strengths.[7] We encouraged residents to share any of their own successful strategies with the group. The session concluded with an exercise designed to help combat feelings of imposter syndrome. We instructed residents to reflect individually on two prompts for approximately 5 minutes: a prior success when they had an initial fear of failure and the positive traits helping them achieve that success, as well as how they would build up their spirits before a difficult task. After time for individual reflection, we offered another opportunity for large-group sharing of responses. We reminded residents of the appropriate steps to take should feelings of imposter syndrome be present in themselves or a colleague. These steps included sharing experiences with a peer, reaching out to a senior resident or faculty member, and seeking counseling through the employee assistance program. Finally, we encouraged participants to share one take-home point with the group. At the conclusion of the session, we provided attendees with a handout (Appendix B) featuring take-home points and quotes from notable individuals on their own experiences with imposter syndrome.

Evaluation

Participants completed an optional postsession survey (Appendix C). The session creators (including faculty, residents, and a psychologist) developed this survey to assess learning objectives and determine whether our session was an effective intervention to promote resident wellness. Answers to survey questions were scored on a 5-point Likert scale, and three open-ended questions elicited qualitative feedback: "What did you like the most about wellness report?" "What can we do to improve future wellness reports such as this?" "Do you have suggestions for future wellness reports?"

Copyright © 2020 Baumann et al. This is an open-access publication distributed under the terms of the Creative Commons Attribution license.

Table 1. Imposter Syndrome Postsession Likert-Scale Survey Results

Question	No. (%)				
	Strongly Disagree	Disagree	Neutral	Agree	Strongly Agree
This was an effective intervention to promote resident wellness. ($n = 21$)	0 (0)	3 (14)	1 (5)	16 (76)	1 (5)
The facilitator was helpful in fostering discussions. ($n = 21$)	0 (0)	0 (0)	1 (5)	15 (71)	5 (24)
I feel comfortable recognizing imposter syndrome in myself. ($n = 21$)	0 (0)	0 (0)	1 (5)	14 (67)	6 (29)
I feel comfortable recognizing imposter syndrome in my colleagues. ($n = 21$)	1 (5)	4 (19)	5 (24)	11 (52)	0 (0)
I feel comfortable discussing imposter syndrome with my colleagues. ($n = 21$)	1 (5)	0 (0)	3 (14)	16 (76)	1 (5)
I know what to do next if myself or my colleague has imposter syndrome. ($n = 21$)	1 (5)	1 (5)	6 (29)	13 (62)	0 (0)
I would get more out of wellness report if it were given during clinic block instead of inpatient firm. ($n = 19$)	0 (0)	3 (16)	7 (37)	8 (42)	1 (5)

Results

Internal medicine residents from all 3 years of training attended the small-group discussion session. We conducted the curriculum on two separate occasions to capture different groups of residents. Both sessions took place in August during an afternoon academic period. A total of 21 attendees completed our optional postsession survey. Two of the 21 residents left the final Likert-scale question unanswered, and many residents refrained from answering the free-response questions.

Table 1 outlines results from the Likert-scale portion of our survey. After completing the session, 96% of residents felt comfortable recognizing imposter syndrome in themselves. Eighty-one percent of residents felt comfortable discussing imposter syndrome with their colleagues, but only 52% felt comfortable recognizing imposter syndrome in their colleagues. Sixty-two percent of attendees knew the appropriate next steps after identifying imposter syndrome in themselves or in a colleague. Eighty-one percent of residents felt that the imposter syndrome wellness session was an effective intervention to promote resident wellness, and 76% thought the facilitator was helpful in fostering discussion. Forty-seven percent of residents felt that they would gain more out of a wellness session during an outpatient block.

Table 2 displays the results from the free-response portion of our survey. Numerous residents commented that they appreciated the open-discussion format and encouraged having residents from all levels of training present. Participants noted that this session was well timed with the transition of trainees into new roles. When residents were asked what they liked the most about wellness report, one stated that "the topic was perfect for this time of year as we each transition to a new role with new responsibilities that may make us uneasy." Areas of improvement identified through free response included offering food, which was suggested by 62% of residents answering the question "What can we do to improve future wellness reports such as this?" Also, a participant suggested placing additional emphasis on what to do if one identifies feelings of imposter syndrome.

Discussion

Through a collaboration with faculty, residents, and psychologists, we developed a highly interactive session for internal medicine residents on imposter syndrome as part of our larger resident wellness curriculum. Almost all participants felt comfortable recognizing imposter syndrome in themselves, while a smaller percentage of residents felt comfortable recognizing imposter syndrome in their colleagues. Many residents felt comfortable discussing imposter syndrome with their peers, but only 62% stated that they knew the next steps to take if imposter syndrome was identified. Participants also commented that they liked the open discussion format and felt our facilitator was helpful in

Table 2. Imposter Syndrome Postsession Survey Free Responses

Question	Free-Response Answers
What did you like the most about wellness report?	"I always appreciate discussion."<br>"Good discussion, good topic for early in the year."<br>"Discussion."<br>"Open discussion format."<br>"Concise!"<br>"Open discussion."<br>"Nice to see the class."<br>"Good questions; liked mixed with interns."<br>"The topic was perfect for this time of year as we each transition to a new role with new responsibilities that may make us uneasy or feel the imposter syndrome."
What can we do to improve future wellness reports such as this?	"Knowing the topic in advance."<br>"Food."<br>"Keep to 30 min."<br>"Have food."<br>"Food; more emphasis on what to do versus recognizing it."<br>"Bring food."<br>"This would be good in groups of six. Two interns, two JARs [second-year residents], two SARs [third-year residents]."
Do you have suggestions for future wellness reports?	"Preventing burnout."<br>"Puppies, pet therapy."

Copyright © 2020 Baumann et al. This is an open-access publication distributed under the terms of the Creative Commons Attribution license.

fostering discussion. Timing of this session in the beginning of the academic year was key to capturing residents during a vulnerable period of transition to new roles. The inclusion of junior and senior residents along with interns provided multiple perspectives on the training environment. Our residents viewed discussion of imposter syndrome as an effective intervention to promote resident wellness and resiliency. Given the connection between imposter syndrome and burnout,[2,3] this topic is a vital component of resident wellness initiatives. We believe that development of the skills to combat imposter syndrome during residency could help mitigate burnout in trainees throughout their medical careers.

As with any new curriculum, we faced obstacles during our implementation and identified several opportunities for improvement. First, it was difficult to find dedicated time for residents to focus on a wellness topic. We utilized existing educational time during inpatient rotations but captured only a small number of residents in these sessions relative to our large program size. Though attendance was both expected and highly encouraged during educational time, sessions were not mandatory. Inpatient rotations are more clinically demanding, and it was challenging for residents to fully invest in self-reflection while receiving calls and messages regarding their patients. Some residents did not attend the full session, and there are likely some residents who attended our session and did not complete a survey. Lunch was not provided, and much of the open-ended feedback we received from attendees related to having food present.

Our curriculum and its evaluation have several limitations. It should be noted that while there appears to be a correlation between imposter syndrome and burnout,[2,3] there is no known causal relationship. There is also no evidence that discussions of imposter syndrome improve burnout in internal medicine residents. We did not collect presession data regarding the participants' baseline knowledge of imposter syndrome, so we unfortunately do not have any comparison for our postsession data. This hindered our ability to fully assess our session objectives. At the end of our session, only 62% of participants commented that they knew what to do if they identified feelings of imposter syndrome in themselves or a peer. We also received a suggestion from one of the residents that the session should focus "on what to do versus recognizing it [imposter syndrome]." It is possible that the discussion-based format of our session, which emphasized helping residents feel safe in identifying and externalizing their feelings of imposter syndrome, may have detracted from the important goal of educating residents on what next steps to take should these feelings arise. Another limitation of our intervention is that attendance was not enforced. As a result, the number of residents who attended the full session and completed the survey was low relative to our program size. Data may be skewed due to self-selection bias as it is possible that residents predisposed to enjoying wellness discussions attended the sessions while residents who did not enjoy discussions avoided it. We were fortunate to have a clinical psychologist lead one of our sessions but recognize that this resource may not be available at all programs. Our second session was conducted successfully by a chief resident. Our facilitator guide (Appendix A) provides a detailed outline of our session with suggested facilitation techniques to aid in successful discussion. Another limitation of our curriculum is the inherent variability of a discussion-based format that relies on facilitator and resident participation. It is impossible to exactly duplicate this curriculum as its success relies heavily on individual participation and group dynamics.

Having determined that our residents found these sessions useful for promoting resident well-being, we would like to improve upon and expand this curriculum. We plan to redesign our survey to include a presession component to appropriately assess knowledge and skills gained during the session. We received important feedback from our participants through the postsession survey on how to improve future sessions. We plan to move these discussions into the protected ambulatory didactic space to improve attendance, allow dedicated time for discussion and self-reflection, and enhance survey data collection. We will provide food if sessions occur around lunch to prevent hunger as a distraction and to save time for residents.

Our postsurvey data revealed that our curriculum was not as successful as we had hoped in an important area: ensuring residents know what to do if imposter syndrome develops. We feel that recognizing and sharing feelings of imposter syndrome with peers through discussion sessions like these are valuable ways to address imposter syndrome. Future facilitators could consider dedicating additional time to the discussion of next steps to ensure that all aspects of this topic are well communicated to participants. Another area of growth for our curriculum on imposter syndrome includes involving medical students. Targeting interventions to medical students may provide tools to address imposter syndrome before the stressful and demanding years of residency. Given the higher prevalence of imposter syndrome in women, those underrepresented in medicine, and international medical graduates, another focus could be the development of discussions specifically on imposter

Copyright © 2020 Baumann et al. This is an open-access publication distributed under the terms of the Creative Commons Attribution license.

syndrome with these groups. A final area of future research includes assessing whether attendance at a session on imposter syndrome affects the development of burnout throughout residency.

In conclusion, short discussion-based sessions on imposter syndrome provided a safe learning environment for residents to discuss the prevalence and presentation of imposter syndrome and strategies to overcome it. Most residents felt comfortable identifying imposter syndrome in themselves and their colleagues, were confident in pursuing help if imposter syndrome was identified, and saw the session as an effective intervention to promote their wellness. Our sessions can easily be implemented as they are less than an hour in length. Residency programs in all disciplines should consider integrating similar discussions on imposter syndrome into future wellness initiatives.

Appendices

A. Imposter Syndrome Facilitator Guide.docx

B. Imposter Syndrome Handout.docx

C. Imposter Syndrome Survey.docx

All appendices are peer reviewed as integral parts of the Original Publication.

Natalie Baumann, MD: Resident Physician, Department of Medicine, Washington University School of Medicine in St. Louis

Carol Faulk, MD: Instructor in Medicine, Department of Medicine, Washington University School of Medicine in St. Louis

Jessica Vanderlan, PhD: Clinical Instructor, Department of Psychiatry, Washington University School of Medicine in St. Louis

Justin Chen, MD: Chief Resident in Quality and Patient Safety, Department of Medicine, John Cochran VA Medical Center

Rakhee K. Bhayani, MD: Associate Program Director, Department of Medicine, Washington University School of Medicine in St. Louis

Disclosures
None to report.

Funding/Support
None to report.

Ethical Approval
Reported as not applicable.

References

1. Accreditation Council for Graduate Medical Education. *ACGME Common Program Requirements*. Accreditation Council for Graduate Medical Education; 2017. Accessed June 7, 2019. https://www.acgme.org/Portals/0/PFAssets/ProgramRequirements/CPRs_2017-07-01.pdf
2. Villwock JA, Sobin LB, Koester LA, Harris TM. Impostor syndrome and burnout among American medical students: a pilot study. *Int J Med Educ*. 2016;7:364-369. https://doi.org/10.5116/ijme.5801.eac4
3. Legassie J, Zibrowski EM, Goldszmidt MA. Measuring resident well-being: impostorism and burnout syndrome in residency. *J Gen Intern Med*. 2008;23(7):1090-1094. https://doi.org/10.1007/s11606-008-0536-x
4. Clance PR, Imes SA. The impostor phenomenon in high achieving women: dynamics and therapeutic interventions. *Psychotherapy (Chic)*. 1978;15(3):241-247. https://doi.org/10.1037/h0086006
5. Oriel K, Plane MB, Mundt M. Family medicine residents and the impostor phenomenon. *Fam Med*. 2004;36(4):248-252.
6. LaDonna KA, Ginsburg S, Watling C. "Rising to the level of your incompetence": what physicians' self-assessment of their performance reveals about the imposter syndrome in medicine. *Acad Med*. 2018;93(5):763-768. https://doi.org/10.1097/ACM.0000000000002046
7. Chandra S, Huebert CA, Crowley E, Das AM. Impostor syndrome: could it be holding you or your mentees back? *Chest*. 2019;156(1):26-32. https://doi.org/10.1016/j.chest.2019.02.325
8. Ramsey JL, Spencer AL. Interns and imposter syndrome: proactively addressing resilience. *Med Educ*. 2019;53(5):504-505. https://doi.org/10.1111/medu.13852
9. Tom Hanks says self-doubt is "a high-wire act that we all walk." NPR. April 26, 2016. Accessed June 19, 2019. https://www.npr.org/2016/04/26/475573489/tom-hanks-says-self-doubt-is-a-high-wire-act-that-we-all-walk
10. Pinker S. *The Sexual Paradox: Men, Women, and the Real Gender Gap*. Scribner; 2008.
11. Bravin J. Memoir details justice's difficult ascent. *Wall Street Journal*. Updated January 14, 2013. Accessed September 17, 2020. https://www.wsj.com/articles/SB10001424127887324595595704578239760608699742

Received: August 19, 2019
Accepted: June 16, 2020
Published: November 10, 2020

Copyright © 2020 Baumann et al. This is an open-access publication distributed under the terms of the Creative Commons Attribution license.
